# Investigating the Effect of Gaze Cues and Emotional Expressions on the Affective Evaluations of Unfamiliar Faces

**DOI:** 10.1371/journal.pone.0162695

**Published:** 2016-09-28

**Authors:** Todd Larson Landes, Yoshihisa Kashima, Piers D. L. Howe

**Affiliations:** School of Psychological Sciences, 12^th^ Floor Redmond Barry Building, University of Melbourne, VIC 3010, Australia; Tilburg University, NETHERLANDS

## Abstract

People look at what they are interested in, and their emotional expressions tend to indicate how they feel about the objects at which they look. The combination of gaze direction and emotional expression can therefore convey important information about people’s evaluations of the objects in their environment, and can even influence the subsequent evaluations of those objects by a third party, a phenomenon known as the emotional gaze effect. The present study extended research into the effect of emotional gaze cues by investigating whether they affect evaluations of the most important aspect of our social environment–other people–and whether the presence of multiple gaze cues enhances this effect. Over four experiments, a factorial within-subjects design employing both null hypothesis significance testing and a Bayesian statistical analysis replicated previous work showing an emotional gaze effect for objects, but found strong evidence that emotional gaze cues do not affect evaluations of other people, and that multiple, simultaneously presented gaze cues do not enhance the emotional gaze effect for either the evaluations of objects or of people. Overall, our results suggest that emotional gaze cues have a relatively weak influence on affective evaluations, especially of those aspects of our environment that automatically elicit affectively valenced reactions, including other humans.

## Introduction

Humans are experts at interpreting the non-verbal signals produced by others [1, 2]. Among the many ways in which we signal with our bodies, the direction of our gaze–which indicates the object, person or location that we are currently interested in—appears to be a particularly salient cue [3, 4]. Recent research suggests that paying attention to others’ gaze cues can do more than simply alert us to the existence of objects in our environment that we have previously overlooked; it can affect how much we like those objects [3, 5–8].

The importance of gaze cues and the ability to interpret them is reflected in a wide range of evidence. The ability to detect and follow another’s gaze is present from infancy and contributes to the development of joint attention, in which the infant (or adult) following the other’s gaze is aware that both are focussing on the same object in the environment [9–11]. Both humans and primates appear to have specialised brain areas for the processing of gaze information, including the superior temporal sulcus [10, 12–14]. Recent studies indicate that adult patients with cortical blindness (e.g., due to destruction of the primary visual cortex) can detect both gaze direction and emotional expressions through subcortical structures including the amygdala, suggesting a prominent evolutionary role for processing emotion and gaze cues [15–17].

In combination, this evidence suggests that the ability to follow and decode others’ gaze cues is a crucial aspect of social life [18]. Despite this, there is little direct evidence of how gaze cues affect our evaluations of the most important aspect of our social environment–other people. The present study aimed to address this gap in the literature by examining how gaze cues and the emotional expressions that sometimes accompany them influence our initial impressions of others.

## Gaze cues orient attention and influence affective evaluations of objects

Early work on gaze cues examined their effects on the orientation of attention. Friesen and Kingstone [19] found that participants were quicker to detect a target (a letter) when it was presented on the side of the screen at which a nonpredictive (valid 50% of the time), emotionally neutral, centrally presented cue face had gazed. This finding has proved to be highly robust, and there is now a large body of evidence that people orient attention automatically in response to gaze cues, both overtly (e.g., measured by saccades [20–24]) and covertly (e.g., measured using reaction time data [3, 5, 25–27]). Participants appear to be unable to suppress their responses to gaze cues even when they are instructed that the cues are counterpredictive (i.e., when the target will appear in the non-cued location more often than in the cued location [22, 28]. Galfano et al. [29] demonstrated this inability to suppress particularly clearly by observing a gaze cueing effect even after participants were told with 100% certainty where the target would appear before the presentation of a gaze or arrow cue.

Interestingly, while one might expect gaze direction to be a particularly salient cue given its biological significance, evidence from the gaze cueing literature indicates that symbolic cues such as arrows orient attention in a very similar fashion, including when they are counterpredictive [22, 23, 29–31]; though cf. [28]. Results using neuroimaging techniques are also equivocal; while some studies report evidence that gaze and arrow cues are processed by distinct networks [32], others have found substantial overlap [33]. Birmingham, Bischof and Kingstone [34] suggest that one way to distinguish between the effects of gaze and arrow cues is to examine which form of spatial cue participants attend to when both are embedded in a complex visual scene. The authors had participants freely view street scenes that included both people and arrows, and found a strong tendency for participants to orient to people’s eye regions rather than arrows.

Another extension of the gaze cueing paradigm which suggests that people might process gaze cues differently than symbolic cues comes from Bayliss et al. [3], in which participants had to classify laterally presented common household objects (e.g., a mug, a pair of pliers). A photograph of an emotionally neutral face served as a central, nonpredictive cue. Bayliss et al. [3] observed the standard gaze cueing effect; participants were quicker to classify those objects that had been gazed at by the cue face. In addition, they asked participants to indicate how much they liked the objects, and found that those objects that were consistently looked at by the cue face received higher ratings than uncued objects. Arrow cues, on the other hand, produced a cueing effect on reaction times, but had no effect on object ratings. This “liking effect” has since been replicated in a number of similar experiments [6–8]. Together, these findings suggest that we might seek out and orient ourselves in response to the gaze of others in part because gaze cues help us “evaluate the potential value of objects in the world” (p. 1065) [3].

## The role of emotional expressions

The superior temporal sulcus, which is thought to be involved in processing both gaze direction [12, 35, 36] and emotional expression [37, 38], is highly interconnected with the amygdala, which is also involved in processing both emotions and gaze direction [17, 35, 39, 40]. Behavioural evidence for a possible link between processing of gaze cues and emotional expressions comes from studies using Garner’s [41] dimensional filtering task. A number of studies have shown that in certain circumstances (e.g., depending on how difficult to discriminate each dimension is), processing of gaze direction and emotional expression interfere with each other [40, 42–44].

Despite the foregoing, studies investigating the interaction between gaze cues and emotional expressions in the attention cueing paradigm have generated mixed evidence. In a comprehensive series of experiments, Hietanen and Leppanen [27] tested whether cue faces expressing different emotions (cue faces were photographs of neutral, happy, angry, or fearful faces) would lead to differences in attentional orienting. They found that attentional cueing effects were similar regardless of emotional expression. A number of other studies have also failed to find any modulation of reaction times by the interaction of gaze cue and emotional expression (see, e.g., Bayliss et al. [5], who found no difference in cueing comparing happy and disgusted cue faces; Galfano et al. [45], who used fearful, disgusted and neutral cues; and Holmes, Mogg, Garcia, & Bradley [46] and Rigato et al. [47], who used neutral, fearful and happy cues). The failure to observe any significant influence of emotion on gaze cueing effects is particularly puzzling in relation to fearful expressions, because both theory and some empirical findings suggest that people should be especially responsive to stimuli that signal a potential threat in the environment (the *behavioural urgency hypothesis* [48–50]). Strengthening the evidence against the application of the behavioural urgency hypothesis to the gaze cueing paradigm, both Galfano et al. [45] and Holmes et al. [46] reported no significant enhancement of cueing by fearful gaze even among participants measuring higher in trait anxiety.

However, other studies have found enhanced cueing effects for fearful gaze cues (compared to happy or neutral cues) among subsets of participants high in trait fearfulness and anxiety [51–53]; still others have shown that participants are more responsive to fearful gaze cues in certain experimental contexts. For example, Kuhn et al. [49] showed that when fearful cue faces appear only rarely (in this experiment, on two trials out of every 97), they do enhance attentional orienting compared with (equally rare) happy cue faces. The nature of the stimuli and the evaluative context of the task also appear to be important. There is evidence that people orient more quickly in response to fearful cues when target stimuli include threatening items, like snarling dogs [54, 55]. Pecchinenda, Pes, Ferlazzo and Zoccolotti [56] reported stronger cueing effects of fearful and disgusted (compared to neutral and happy) cue faces when participants were asked to rate target words as positive or negative; however, when the task was simply to determine whether the letters of the target words were upper- or lower-case, the cue face’s emotion had no impact on gaze cueing effects.

Further evidence that experimental context affects how participants process emotional gaze cues comes from Bayliss et al. [5]. In this extension of Bayliss et al. [3], participants were asked to rate kitchen and garage items that had been consistently cued or gazed away from by emotionally expressive cue faces. The authors did not observe any difference in cueing effects (measured by reaction time) for happy versus disgusted cue faces; there was, however, an interaction when it came to object ratings, with objects cued with a happy expression receiving the highest ratings, objects cued with a disgusted expression receiving the lowest ratings, and uncued objects being rated in between regardless of the cue face’s emotion. This interaction indicates that participants integrated gaze cues with emotional expressions when they were evaluating target objects. Bayliss et al. [5] reported a larger liking effect than Bayliss et al. [3], suggesting that emotionally expressive gaze cues enhanced the liking effect compared with neutral gaze cues. This makes intuitive sense; for example, one would expect a happy gaze towards an object to be a stronger signal of liking than a neutral gaze.

Together, the findings outlined above suggest that the human response to gaze cues is sophisticated and complex, and that careful experimental design is necessary to uncover the subtleties of the process. If a cue face’s emotional expressions are meaningless in an experimental paradigm, one should not necessarily expect them to have any effect; likewise, if an experiment is devoid of any social context, arrow cues appear to orient attention just as strongly as gaze cues [34, 54]. While researchers have begun to elucidate how contextual details such as the nature of stimuli and the meaningfulness of emotion influence orientation of attention in response to gaze cues, there is still much room for exploration of how similar contextual details might affect the way in which gaze cues influence evaluations.

## The effect of gaze cues on evaluations of other people

As noted above, a number of studies have replicated Bayliss and colleagues’ findings that gaze cues can influence participants’ affective evaluations of objects. However, the majority of this work has employed both neutral cue faces and target stimuli; for example, stimuli have included common household objects [3, 5, 57]; paintings specifically chosen for their neutrality [58]; alphanumeric characters [7]; and unknown brands of bottled water [8]; and, with the exception of Bayliss et al. [5], each of these studies used emotionally neutral cue faces. In the present study, we sought to extend this work by examining the influence of gaze cues on evaluations of *other people*; that is, we were interested in testing whether seeing a cue face gaze towards a target face with a positive expression would result in that target face being considered more likeable than a target face gazed at with a negative expression.

There is reason to think that faces might be less susceptible to a liking effect than the neutral stimuli discussed above. Unlike mugs and bottled water, faces evoke strong, affectively valenced evaluations automatically. Willis and Todorov [59] have shown that stable inferences about traits such as attractiveness, likeability, trustworthiness and competence are made after exposure to unfamiliar faces of only 100 milliseconds. In these circumstances, the effect of gaze cues might be undetectable unless they are quite large. However, there is evidence to suggest that evaluations of affectively valenced items and other people can be influenced by gaze cues. Soussignan et al. [60] found that gaze cues from emotionally expressive cue faces (joyful, neutral, and disgusted) had a small effect on ratings of familiar food items. Like faces, food automatically triggers valenced evaluations; the “pleasantness” of food products is automatically processed and is linked to autonomic processes such as mouth-watering and lip-sucking [61, 62]. Jones et al. [63] reported that evaluations of other people are influenced by emotional gaze cues in the context of mate selection. In that study, two male target faces were presented in each trial; a female cue face gazed towards one of them with a positive expression, and ignored the other. Participants were then asked to indicate which of the two target faces they found more attractive. Female participants rated a man who had been smiled at by a female cue face as more attractive than a man who had been ignored; male participants showed the opposite effect. Jones et al. [63] suggested that female participants were exhibiting “mate choice copying effects,” while males were responding to within-sex competition (p. 899).

There are also theoretical reasons to expect that emotional gaze cues affect evaluations. Accurately evaluating other people is very important; aligning ourselves with others who are uncooperative is costly, as is the failure to identify likely collaborators [64, 65]. It would therefore make sense for people to use all potential sources of information–including emotional gaze cues–when evaluating other people so as to make the best possible decisions. Evidence from the gossip literature shows that people do indeed use information about others to update their evaluations and guide behaviour [66–68].

For these reasons, we predicted that emotional gaze cues would affect participants’ evaluations of novel others in the present study. That is, we expected to observe an interaction effect between the cue face’s emotional expression and gaze direction, such that target faces looked at with a positive expression would be liked more than target faces looked at with a negative expression, and that target faces that were looked away from would receive medium ratings, regardless of the cue face’s emotional expression (Hypothesis 1). Our method was largely similar to that of Bayliss et al. [5], with the exception of a multiple cue face condition (discussed below), and the expressions of our cue faces. Because the stimuli we used were not threatening or disgusting, fearful and disgusted cue faces were not appropriate. Most relevant to the evaluative context of our experiment were expressions of liking and disliking; as such, cue face models were instructed to pretend that they were looking at someone they liked and were happy to see for the positive expression, and to pretend they were looking at someone they disliked and mistrusted for the negative expression. Unlike Jones et al. [63], our target faces were diverse in gender and age, and participants rated how much they liked target faces individually rather than comparing their attractiveness.

Given our experimental context (i.e., no threatening or aversive stimuli, no fearful or disgusted cue faces), we did not expect to see an effect of emotion on reaction times; however, reaction times were recorded and tested for a standard gaze cueing effect (i.e., faster reaction times at cued locations) as a manipulation check.

## The effect of multiple cue faces

We also integrated recent developments in the gaze cueing literature by including a multiple cue face condition. As Capozzi et al. noted [57], a single person’s positive attitude towards an object conveys information of somewhat limited value. The object might indeed be generally desirable, or it might be desirable only to the individual observing it for idiosyncratic reasons. When a number of people react in the same way, however, their consensus is more likely to provide valuable information. To test this, Capozzi et al. [57] modified the gaze cueing paradigm of Bayliss et al. [3] such that some objects were consistently cued by the same emotionally neutral face (the single cue condition), while other objects were cued by a different cue face in each of the task’s seven blocks (the multiple cue condition). Capozzi et al. [57] found that gaze cues exerted a stronger effect on evaluations in the multiple cue condition.

In the present study, we extended the work of Capozzi et al. [57] in two ways. Firstly, we examined the effect of gaze cues using emotionally expressive rather than neutral cue faces. Secondly, in order to reduce the memory burden on participants and enable them to more clearly distinguish between the single and multiple cue conditions, our multiple cue face condition involved presenting the multiple cues faces simultaneously rather than individually in separate blocks. In line with Capozzi et al. [57], we expected this emotional gaze effect to be stronger when there were multiple cue faces (Hypothesis 2).

## Experiment 1

### Method

This research was approved by the Psychological Sciences Human Ethics Advisory Group (HEAG) at the University of Melbourne (Ethics ID: 1543939). All participants gave written consent to participate in the experiment after reading a 'Plain Language Statement' outlining the nature of the experiment in a manner approved by the HEAG. Participants were tested for normal or corrected-to-normal vision and received course credit for participating.

Participants were first year undergraduate students in the School of Psychological Sciences at the University of Melbourne, some of whom may not have turned 18. These students were considered competent to give informed consent given that the experiments were simple with no known risks. This procedure was approved by the HEAG. Participants for all subsequent experiments were recruited in the same way.

#### Participants

Thirty-six participants (32 females) with a mean age of 18.8 years (*SD* = 1.12, range = 17–22 years) were recruited for this experiment.

#### Apparatus and stimuli

Stimuli presentation and data collection took place in a lab containing 12 PCs. Participants were seated approximately 60 cm away from the screen, with refresh rate set at 70 hertz.

**Fig 1:**
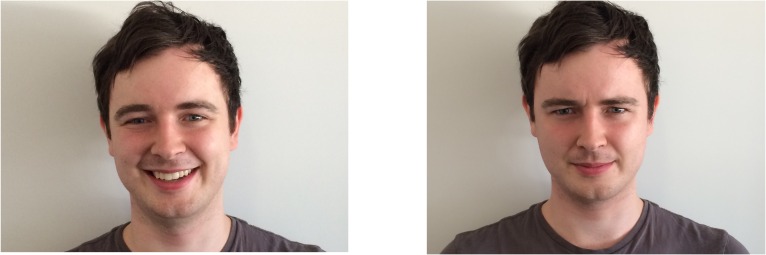
Cue face emotional expressions. Cue face exhibiting a positive (left) and negative (right) expression. All individuals whose images are published in this paper gave written informed consent (as outlined in PLOS consent form) to the publication of their image.

Photographs (dimensions were 9.8 degrees of visual angle (°) x 10.2°) of three males aged 21 to 24 were used as cue faces. There were five versions of each cue face: looking straight ahead with a neutral expression; looking left and right with a positive expression; and looking left and right with a negative expression (Fig 1).

Where cue faces were directing their gaze to one side, the whole head was turned (i.e., the orientation of the head as well as eye gaze indicated direction of gaze). This was to ensure that there was no ambiguity about where the cue face’s attention was directed [63]. All male cue faces were used for consistency. While there is evidence that females respond more strongly to gaze cues than males, no studies that we are aware of indicate that the gender of the cue face modulates the gaze cueing effect [69–71].

Target faces (14.9° x 10.6°) were taken from a database of facial photographs compiled by Bainbridge, Isola, and Oliva [72]. Sixty-eight male and 68 female faces that had received average (from 4 to 6 on a 9-point Likert-type scale) ratings on attractiveness and trustworthiness in Bainbridge et al.’s [72] study were selected as target faces. Attractiveness and trustworthiness are particularly highly correlated with judgments of likeability [73, 74]; as such, we selected for average ratings on these traits to avoid floor and ceiling effects on likeability and maximise the possibility of observing a gaze cueing effect. All target faces had a neutral expression and were gazing at the camera. Ages of target faces ranged from 20 to 60 years. In order to facilitate categorisation of the target faces, a letter (either “x” or “c” in size 14 lowercase font) was superimposed between the eyes using the image manipulation program “GIMP”. This method of categorisation was chosen because we considered that categorising by an inherent characteristic such as sex, age, or race might prime in-group/out-group biases that would introduce additional noise into the data, making any effect of gaze cueing more difficult to detect [75, 76].

#### Design

There were three within-subjects factors, each with two levels. The *gaze cue* factor manipulated the cue face’s gaze direction; in the cued condition, the cue face looked toward the target face, while in the uncued condition the cue face looked away from the target face, toward the empty side of the screen. The *emotion* factor was the manipulation of the cue face’s emotional expression (either positive or negative). The *number of cues* factor was the single or multiple cue face manipulation. There was one cue face in the single cue face condition. All three cue faces were presented in the multiple cue face condition. Finally, the primary dependent variable was the participants’ affective evaluations of the target faces on a nine point scale. Reaction times were also measured to ensure that participants were completing the task as instructed.

#### Procedure

Participants were instructed to ignore the nonpredictive cue face and indicate (by pressing the “x” or “c” key on the keyboard) as quickly as possible whether the target face had an “x” or “c” on it. Framing the task as a measure of reaction time was intended to obscure the study’s hypotheses from participants [3, 5].

**Fig 2:**
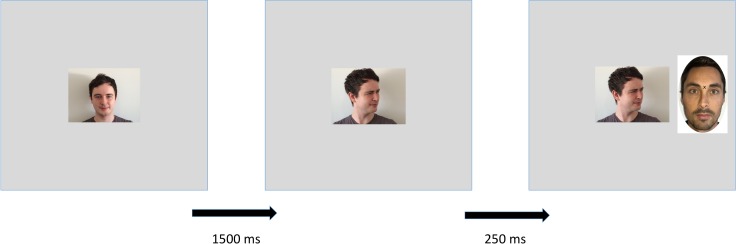
Categorisation trial. Example of a categorisation trial in which a single cue face gazes at a target face with a negative expression.

For each trial of the categorisation task, the cue face first appeared in the centre of the screen gazing straight ahead with a neutral expression for 1500 ms. It then turned to the left or right with either a positive or negative emotional expression for 250 ms before the target face appeared to one side of the screen. The cue and target faces then remained on screen until the participant’s response (Fig 2). After response, participants were given feedback as to the correctness of their answer, and asked to press any key to begin the next trial. Participants were informed of the number of trials remaining in each block.

After receiving instructions, participants completed a practice block of four trials, which were not included in the analysis. They then did two blocks of 64 trials each of the categorisation task, where all 64 target faces not used in the practice trial were displayed once in randomised order. Target faces were displayed under the same cueing, emotion, and number of cue conditions each of the three times they appeared to ensure robust encoding of target faces and cueing conditions [5]. The same cue face was used for each single cue face trial throughout the task. Selection of this “main” cue face was counterbalanced across participants.

The rating block followed the two categorisation blocks. In this block, after categorising each target face, participants were presented with a column of numbers from 9 (at the top of the screen) to 1 (at the bottom of the screen), with the message “How much did you like that person?” at the top of the screen. The prompt “Like very much” was just above the 9, and the prompt “Didn’t like at all” was just below the 1. Reaction times were not collected in the rating block. The entire experiment took approximately 30 minutes to complete.

### Results

As hypotheses were clearly directional and based on previous research, and there was no theory to suggest that effects in the unpredicted direction might be observed, one-tailed tests were used [77–79]. Two-tailed tests were used for all effects not pertaining to the hypotheses. Although the *F* distribution is asymmetrical, this does not prevent the use of a one-tailed test; it simply requires adjusting the *p* value to reflect the probability of correctly predicting the direction of an effect [79–81].

Raw data for this experiment can be found in supporting information file S1 Experiment 1 Dataset.

#### Reaction times

Reaction times were analysed using a within-subjects ANOVA. There was evidence of moderate positive skew in the data (maximum ratio of skewness to standard error = 5.1). However, ANOVA is generally robust to skew when means come from distributions with similar shapes [82, 83]. As this was the case here, no transformation was undertaken. This mirrors the approach taken in previous studies in the gaze cueing literature [3, 5, 19, 27]. Average reaction times were calculated using only data from trials in which the correct classification decision was made. Participants were generally accurate (error rate was 5.9%), and there was no effect of the within-subjects factors on error rates.

Results of a within-subjects ANOVA with reaction time as the dependent measure are shown in Table 1. As expected, there was a main effect of gaze cue, but no evidence of a main effect of emotion or an emotion by gaze cue interaction. Cued target faces (*M* = 650 ms, *SE* = 14) were classified more quickly than uncued target faces (*M* = 695 ms, *SE* = 14) regardless of the cue face’s emotional expression. Reaction times were also quicker in the multiple cue face condition (*M* = 677 ms, *SE* = 14) than the single cue condition (*M* = 667 ms, *SE* = 13); however, because this did not interact with the gaze cue factor this result simply indicated a general tendency for participants to respond more quickly when there were multiple cues present, regardless of whether the gaze cues were valid or not.

**Table 1:** Results of within-subjects ANOVA on reaction times.

Effect	*F*(1, 35)	*p*	*η*_*p*_^*2*^
**Gaze cue**	**73.25**	**< .001**^#^***	**.68**
Emotion	0.02	.88	< .01
Number of cues (“Number”)	7.82	.008**	.18
Emotion x Gaze cue	0.67	.42	.02
Emotion x Number	0.05	.82	< .01
Gaze cue x Number	0.08	.78	< .01
Gaze cue x Emotion x Number	0.57	.46	.02

# = one-tailed test.

** = significant at alpha = .01.

*** = significant at alpha = .001.

#### Evaluations

Across all cueing conditions, faces received ratings very close to the mid-point of the scale (*M* = 5.12, *SD* = 0.80) and data were approximately normal. A within-subjects ANOVA on ratings showed a significant main effect of emotion, with target faces appearing alongside positive cue faces receiving higher ratings than target faces alongside negative cue faces, *M* = 5.20 (*SE* = 0.11) versus *M* = 5.05 (*SE* = 0.11) (Table 2). There was no main effect of gaze cue or the number of cue faces. The hypothesised emotion x gaze cue interaction was not observed, nor was the emotion x gaze cue x number of cues interaction.

**Table 2:** Results of Within-Subjects ANOVA on Target Face Ratings.

Effect	*F*(1, 35)	*P*	*η*_*p*_^*2*^
Emotion	5.53	.01*	.14
Gaze cue	1.0	.32	.03
Number of cues (“Number”)	0.71	.41	.02
Gaze cue x Number	2.08	.16	.06
Emotion x Number	1.60	.22	.04
**Emotion x Gaze cue (H1)**	**0.59**	**.45**^**#**^	**.02**
**Emotion x Gaze cue x Number (H2)**	**0.07**	**.80**^**#**^	**< .01**

# = one-tailed test.

* = significant at alpha = .05. “H1” and “H2” (in bold) indicate that the effects are the subjects of Hypotheses 1 and 2, respectively.

### Discussion

Neither of our hypotheses were supported. While emotion had a main effect on ratings as has previously been observed [5], this did not interact with the cue face’s gaze direction in the expected manner, nor did the number of cue faces enhance the emotion x gaze cue interaction.

The fact that target faces generally received ratings very close to the mid-point of the scale confirmed that our set of target faces was suitable for the task and that floor and/or ceiling effects were unlikely to be the reason for the failure to observe the hypothesised effects. Likewise, the reasonably low error rate and the strong effect of gaze cues on reaction times indicated that participants were attending to the task and orienting in response to the gaze cues in line with previous research.

In response to these results, a direct replication of Bayliss et al. [5] was undertaken. We reasoned that a successful replication would provide evidence that the null results in Experiment 1 were due to the nature of the target stimuli rather than a more general issue with the replicability of the gaze cueing effect reported by Bayliss et al. [5].

## Experiment 2

### Method

#### Participants

Thirty-six participants (26 females) with a mean age of 19.6 years (*SD* = 1.07, range = 17–23 years) were recruited.

#### Apparatus, stimuli, design and procedure

**Fig 3:**
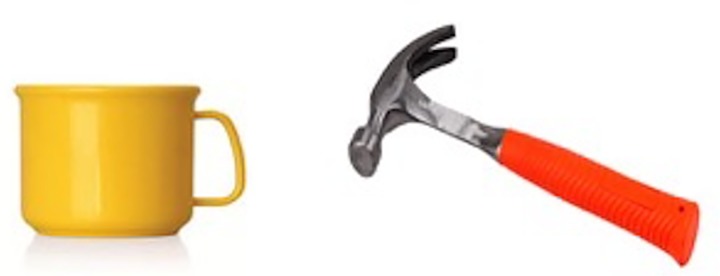
Object stimuli. Examples of a kitchen item (left) and a garage item (right) used as target stimuli in Experiment 2.

The method for Experiment 2 was the same as that for Experiment 1 with minor differences. First, pictures of objects rather than faces were the target stimuli. Following Bayliss et al. [5], thirty-four objects commonly found in a household garage and 34 objects commonly found in the kitchen were used as target stimuli. Pictures of the objects were sourced from the internet (Fig 3). Participants classified the objects as kitchen or garage items by pressing the “k” or “g” buttons on the keyboard. Because it was not necessary for the categorisation task and because this experiment was intended to be a direct replication of Bayliss et al. [5], letters were not superimposed on the objects as was done with the target faces in Experiment 1.

### Results

Data from two participants whose average reaction times were more than three standard deviations slower than the mean were excluded. Exclusion of this data did not change the statistical significance of any of the results reported below.

The approach to data analysis in this experiment and the two that followed was the same as that in Experiment 1. Hypotheses remained the same for all four experiments (though in Experiments 2 and 3 objects were the target stimuli rather than faces). All effects relating to hypotheses were tested with one-tailed tests, while tests of those effects not pertaining to the specific hypotheses were two-tailed. Skew in reaction time data was similar in all four experiments; transformations were not undertaken for the reasons provided above. Finally, error rates were low (from 6.7% to 7.7%) and unrelated to the independent variables in all experiments.

Raw data for this experiment can be found in supporting information file S2 Experiment 2 Dataset.

#### Reaction times

Though objects looked at by the cue face were classified more quickly (mean = 699 ms, *SE* = 18) than those the cue face looked away from (mean = 711 ms, *SE =* 19), a within-subjects ANOVA did not provide evidence to suggest that this difference was significant (see Table 3).

**Table 3:** Results of within-subjects ANOVA for reaction times.

Effect	*F*(1, 33)	*p*	*η*_*p*_^*2*^
**Gaze cue**	**1.97**	**.085**^**#**^	**.06**
Emotion	0.52	.48	.02
Number of cues (“Number”)	0.38	.54	.01
Emotion x Gaze cue	3.24	.08	.09
Emotion x Number	0.45	.51	.01
Gaze cue x Number	0.09	.76	< .01
Emotion x Gaze cue x Number	0.77	.39	.02

# = one-tailed test

#### Evaluations

There was a significant main effect of emotion; objects alongside positive cue faces were rated higher (*M* = 5.29, *SE* = 0.19) than objects alongside negative cue faces (*M* = 4.90, *SE* = 0.18). This was qualified by the predicted two-way interaction between emotion and gaze cue. However, there was no evidence of a three-way interaction between emotion, gaze, and number of cue faces (Table 4).

**Table 4:** Results of Within-Subjects ANOVA on Object Ratings.

Effect	*F*(1, 33)	*p*	*η*_*p*_^*2*^
Emotion	5.08	.03*	.13
Gaze cue	0.03	.87	< .01
Number of cues (“Number”)	0.43	.52	.01
Gaze cue x Number	0.04	.85	< .01
Emotion x Number	0.07	.79	< .01
**Emotion x Gaze cue (H1)**	**3.44**	**.04**^#^*	**.09**
**Emotion x Gaze cue x Number (H2)**	**0.01**	**.94**^**#**^	**< .01**

# = one-tailed test.

* = significant at alpha = .05.

#### Emotion x gaze cue interaction

**Fig 4:**
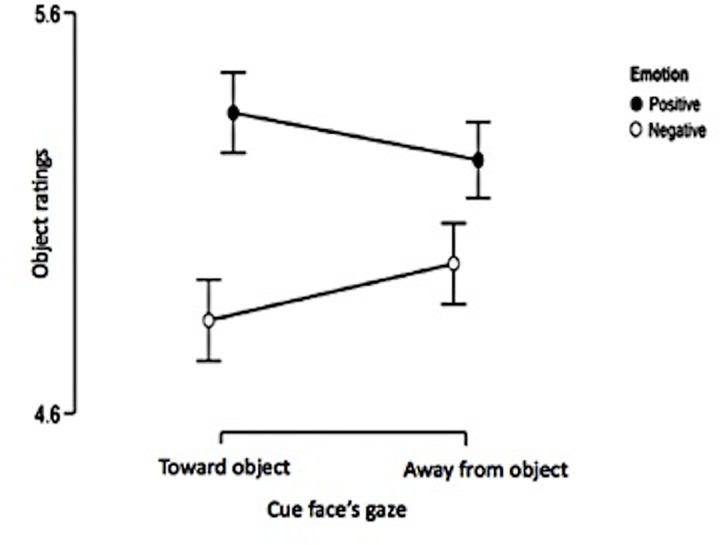
Emotion x gaze cue interaction. Points represent marginal means, bars represent standard errors.

Inspection of sample means showed that the emotion x gaze cue interaction was in the expected direction (Fig 4). As expected, the difference between the emotion expression was significant for the cued objects (*t*(33) = 2.71, *p* = 0.005 (one-tailed), Cohen’s *d* = 0.47) but not for the uncued objects (*t*(33) = 1.43, *p* = 0.16, Cohen’s *d* = 0.25).

### Discussion

The results replicated those of Bayliss et al. [5] with respect to evaluations; participants’ evaluations of the objects were in line with cue faces’ emotionally expressive gaze cues. Interestingly (and unlike Bayliss et al. [5]), this effect of gaze cues on evaluations was seen despite the lack of any significant effect of gaze cues on reaction times. However, counter to Hypothesis 2, there was no evidence that the evaluation effect was strengthened in the multiple cue condition. The successful replication of Bayliss et al.’s [5] finding suggested that the failure to observe an effect of gaze cues on evaluations in Experiment 1 might have been due to the nature of the stimuli. This may have been because stimuli were faces rather than objects. However, it may also have been because target stimuli had letters superimposed on them. Participants in Experiment 1 may have selectively attended to the letters (and not the faces they were superimposed upon) because only the letters were relevant to the categorisation task [84, 85]. Limited processing of target faces might have resulted in the faces being rated more or less at random, or meant that additional information, such as gaze cues, was not integrated when participants encoded the target faces [86]. In order to investigate this possibility, a further experiment was run in which letters were superimposed on objects.

As the effect size of the emotion x gaze cue interaction in Experiment 2 was smaller than that reported by Bayliss et al. [5] (*η*_*p*_^*2*^ = .09 compared with .19), the sample size was increased to 48 participants in Experiments 3 and 4; this was a substantially larger sample size than the 26 recruited by Bayliss et al. [5] or the 28 recruited by Jones et al. [63].

## Experiment 3

### Method

#### Participants

Forty-eight participants (37 females) with a mean age of 20.0 years (*SD* = 5.46, range = 17–45 years) were recruited.

#### Apparatus, stimuli, design and procedure

**Fig 5:**
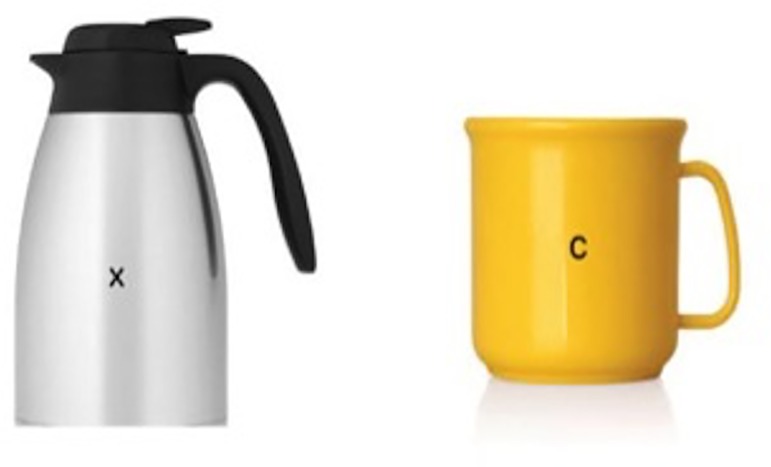
Examples of target objects used in Experiment 3.

The method for Experiment 3 was the same as that for Experiment 2 with one change; objects had letters superimposed on them using the image manipulation program GIMP. As in Experiment 1, the letters “x” and “c” in size 14 lowercase font were used (Fig 5 below). Half of the kitchen objects were marked with an “x” and the other half with a “c”; the same approach was taken with garage objects.

### Results

#### Reaction times

Participants were significantly quicker to classify cued objects (*M* = 637 ms, *SE* = 17) than uncued objects (*M* = 678 ms, *SE* = 17). No other main effects or interactions were significant (see Table 5).

**Table 5:** Results of within-subjects ANOVA on reaction times.

Effect	*F*(1, 47)	*p*	*η*_*p*_^*2*^
**Gaze cue**	**44.65**	**< .001**^#^***	**.49**
Emotion	0.12	.73	< .01
Number of cues (“Number”)	0.14	.71	< .01
Emotion x Gaze cue	1.30	.26	.03
Emotion x Number	0.23	.63	< .01
Gaze cue x Number	2.87	.10	.06
Emotion x Gaze cue x Number	0.76	.39	.02

# = one-tailed test.

*** = significant at alpha = .001.

#### Evaluations

There was a main effect of emotional expression, with positive cue faces eliciting higher ratings (*M* = 5.36, *SE* = 0.11) than negative cue faces (*M* = 5.18, *SE* = 0.13), but no other significant main effects or interactions (see Table 6).

**Table 6:** Results of Within-Subjects ANOVA on Object Ratings.

Effect	*F*(1, 47)	*p*	*η*_*p*_^*2*^
Emotion	5.54	.02*	.11
Gaze cue	0.42	.52	< .01
Number cue faces (“Number”)	2.23	.14	.05
Gaze cue x Number	0.46	.50	.01
Emotion x Number	<0.01	.97	< .01
**Emotion x Gaze cue (H1)**	**0.15**	**.70**^**#**^	**< .01**
**Emotion x Gaze cue x Number (H2)**	**0.54**	**.22**^**#**^	**.01**

# = one-tailed test.

* = significant at alpha = .05.

In order to investigate the influence of the superimposed letters across Experiments 2 and 3, *Experiment* was included as a between-subjects factor in a mixed ANOVA as per Nieuwenhuis et al. [87]. We found that the emotion x gaze interaction effect was not significantly different across experiments (*F*(1,80) = 2.37, *p* = .13, *η*_*p*_^*2*^ = .03) nor was the emotion x gaze cue x number of cues interaction (*F*(1,80) = 0.31, *p* = .58, *η*_*p*_^*2*^ < .01).

Raw data for this experiment can be found in supporting information file S3 Experiment 3 Dataset.

### Discussion

The primary aim of this experiment was to determine whether the letters superimposed on target stimuli might have interfered with the way in which participants processed target stimuli, and thereby nullified the effect of cue faces’ gaze cues. Although the emotion x gaze cue interaction was significant in Experiment 2 and non-significant in Experiment 3, the difference between these two interaction effects was itself not statistically significant [87, 88]. As such, the impact of the superimposed letters on the results of Experiment 1 remains ambiguous. There was also no evidence to suggest that the emotion x gaze x number of cues interaction was affected by the superimposed letters; however, this was of less interest because that interaction had not been significant in either of the first two experiments. Despite the lack of clear evidence about the effect of the superimposed letters, we adopted a conservative approach and repeated Experiment 1 with the potentially problematic letters removed from the target faces.

## Experiment 4

### Method

#### Participants

Forty-eight participants (38 females) with a mean age of 20.3 years (*SD* = 5.72, range = 18–47 years) were recruited.

#### Apparatus, stimuli, design and procedure

The method for Experiment 4 was the same as that for Experiment 1 with one change; target faces did not have letters superimposed on them. Participants classified target faces based on sex using the “m” and “f” keys. Sex was chosen as the characteristic for classification because there is less potential for ambiguity about sex than there is about age or race.

### Results

One participant’s data were excluded due to mean reaction times more than three standard deviations slower than the mean. Exclusion of these data did not change the results of any significance tests.

#### Reaction times

Once again, participants were significantly quicker to react to cued faces (*M* = 590 ms, *SE* = 14) than uncued faces (*M* = 607 ms, *SE* = 14). There was also a main effect of the number of gaze cues, with participants faster to classify faces in the multiple cue face condition (*M* = 591 ms, *SE* = 14 compared with *M* = 606 ms, *SE* = 14 in the single cue face condition). No other main effects or interactions were significant (see Table 7).

**Table 7:** Results of within-subjects ANOVA on reaction times.

Effect	*F*(1, 46)	*p*	*η*_*p*_^*2*^
**Gaze cue**	**12.87**	**< .001**^#^***	**.22**
Emotion	0.05	.82	< .01
Number of cues (“Number”)	11.23	.002	.20
Emotion x Gaze cue	0.09	.77	< .01
Emotion x Number	0.07	.79	< .01
Gaze cue x Number	0.24	.63	< .01
Emotion x Gaze cue x Number	0.19	.67	< .01

# = one-tailed test.

*** = significant at alpha = .001.

Raw data for this experiment can be found in supporting information file S4 Experiment 4 Dataset.

#### Evaluations

There was a main effect of emotional expression, with positive cue faces eliciting higher ratings (*M* = 4.93, *SE* = 0.17) than negative cue faces (*M* = 4.73, *SE* = 0.17), but no other significant main effects or interactions (see Table 8). The emotion x gaze cue interaction was in the expected direction but did not reach statistical significance.

**Table 8:** Results of Within-Subjects ANOVA on Ratings of Target Faces.

Effect	*F*(1, 46)	*P*	*η*_*p*_^*2*^
Emotion	14.00	< .001***	.23
Gaze cue	2.29	.14	.05
Number of cues (“Number”)	0.17	.68	< .01
Gaze cue x Number	0.39	.54	< .01
Emotion x Number	0.29	.59	< .01
**Emotion x Gaze cue (H1)**	**1.53**	**.11**^**#**^	**.03**
**Emotion x Gaze cue x Number (H2)**	**< 0.01**	**.94**^**#**^	**< .01**

# = one-tailed test.

*** = significant at alpha = .001.

A between-subjects comparison across Experiments 1 and 4 was undertaken to determine whether removing the superimposed letters made a difference to the emotion x gaze cue interaction effect when faces were the target stimuli. As with objects, there was no significant difference across experiments, *F*(1, 82) = 2.07, *p* = .15, *η*_*p*_^*2*^ = .03. On this basis, we then combined the Experiment 1 and 4 data sets. Operating on this combined data set we still found no evidence for either an emotion x gaze cue interaction (*F*(1,83) = 0.138, *p =* .71, *η*_*p*_^*2*^ = .002) or an emotion x gaze cue x number interaction (*F*(1,83) = 0.008, *p =* .930, *η*_*p*_^*2*^ < .001).

### Discussion

There was no evidence to suggest that facial evaluations were affected by the gaze cues and emotional expressions of the cue faces. Although the effect was in the expected direction, it was not significantly different from the emotion x gaze cue interaction observed in Experiment 1; as such, there was once again no clear evidence to suggest that the superimposed letters interfered with the gaze cueing effect. There was also no evidence that participants were more affected by the emotion x gaze cue interaction in the multiple cue face condition than they were in the single cue face condition.

## Bayesian Analysis of Null Results

A limitation of null hypothesis significance testing (NHST) is that it does not permit inference about the strength of evidence in favour of the null hypothesis. Bayesian inference does not suffer from this limitation [89, 90]. Given the large number of null findings in the experiments reported here (see Table 9), additional analysis using Bayesian statistics was undertaken in order to quantify the strength of evidence for the null hypothesis. The Bayesian null hypothesis examined here is one of no effect in either direction since we wished to evaluate the level of evidence that there is no effect at all, not just no effect in a particular direction. All null findings were analysed with Bayesian repeated measures ANOVAs using the software platform JASP [91]. A conservative approach was taken by adopting JASP’s uninformative default prior in all analyses [90, 92].

**Table 9:** Summary of Results Across All Four Experiments.

Experiment	Hypothesis 1	Hypothesis 2
1—Faces with letters	N	N
2 –Objects	Y	N
3—Objects with letters	N	N
4 –Faces	N	N

Y = Hypothesis supported by significant result at alpha = .05 (one-tailed); N = Hypothesis not supported. Hypothesis 1: There will be a gaze x emotion interaction. Hypothesis 2: There will be a gaze x emotion x number interaction.

Bayes factors for inclusion (BF_Incs_) were computed to compare the evidence that a hypothesised effect was non-zero with the evidence that the effect was zero (i.e., the null hypothesis). The BF_Incs_ therefore represents the odds ratio in support of the alternative hypothesis relative to the null hypothesis [93]. Conversely, a large 1/ BF_Inc_ represents the odds ratio in support of the null hypothesis relative to the alternative hypothesis. As shown in Table 10, for the data sets of Experiments 1 and 4 combined, the odds ratio for the null hypothesis relative to the alternative hypothesis was 34.5:1, which represents “strong” support for the null hypothesis [91]. This suggests that the emotional gaze effect does not occur for face stimuli. In other words, the likeability of a face is not influenced by the gaze direction and emotional expression of a third party.

**Table 10:** Bayesian analysis of null results in relation to hypothesized gaze x emotion interaction.

Experiment	BF_Inc_	1/ BF_Inc_
1*	0.175	5.71
3*	0.102	9.80
4	0.640	1.56
1* & 4	0.029	34.5

* = experiment in which targets had letters superimposed.

The value for 1/ BF_inc_ indicates support for the null hypothesis.

In relation to Hypothesis 2—that the gaze x emotion interaction will be larger when there are more onlookers—1/BF_Incs_ indicate “extreme” [91] evidence in favour of the null hypothesis that the number of gaze cues had no effect on the emotional gaze effect, regardless of whether those stimuli were faces or objects (Table 11). Across all four experiments, the minimum odds ratio was 323:1 in favour of the null hypothesis.

**Table 11:** Bayesian analysis of null results in relation to the hypothesized gaze x emotion x number interaction.

Experiment	BF_Inc_	1/ BF_Inc_
1*	0.0031	323
2	9.9e-4	1,014
3*	4.3e-4	2,352
4	0.0012	833
1* & 4	1.6e-4	6398

* = experiment in which targets had letters superimposed.

The value for 1/ BF_inc_ indicates support for the null hypothesis.

## General Discussion

### Evaluations

The impact of emotionally expressive gaze cues on the affective evaluations of target stimuli was investigated over four experiments. Although Bayliss et al.’s [5] finding that the affective evaluations of common household objects could be modulated by emotionally expressive gaze cues was replicated in Experiment 2, this effect was not seen when faces were the target stimuli. A follow-up Bayesian analysis of the results from Experiments 1 and 4 found an odds ratio of 34.5:1 in favour of the null hypothesis, indicating that in our experiments the emotional gaze effect did not occur for faces. Similarly, our Bayesian analysis showed that increasing the number of onlookers did not increase the emotional gaze effect for evaluations of either face or object stimuli. Analysis of reaction times suggested that these null results were not due to a failure of the gaze cues to manipulate participants’ attention. Strong gaze cueing effects were observed in three of the four experiments, and the one experiment in which gaze cueing effects were marginal (Experiment 2) was the one in which the evaluation effect was significant.

The pattern of results seen both here and in other work suggests that gaze cues–whether accompanied by emotional expressions or not—are most likely to affect evaluations of mundane, everyday objects that do not automatically elicit valenced reactions. Small- to medium-sized effects of gaze cueing have been reliably observed when target stimuli are affectively neutral objects (e.g., this study’s Experiment 2; see also [3, 5, 8]; though c.f. this study’s Experiment 3 for no effect and Treinen et al. [58] for a larger effect). When stimuli are affectively valenced, however, the effect of gaze cues appears to be weaker. For example, the effect of gaze cues on evaluations of food in Soussignan et al. [60] was smaller than any of the effect sizes reported with neutral stimuli, and the present study failed to demonstrate evidence of a gaze cueing effect on faces. The exception to this trend is Jones et al. [63], in which participants’ evaluations of the attractiveness of target faces *were* influenced by emotionally expressive gaze cues, with effect sizes similar to those seen with neutral objects.

There are important procedural differences between Jones et al. [63] and the broader gaze cueing literature (the present study included). Firstly, Jones et al. [63] investigated the effects of gaze cues in the context of mate selection. A number of authors have suggested that social transmission of mate preferences is a sophisticated process that may differ from transmission of preferences more generally [94, 95]; as such, the results of Jones et al. [63] may not generalise beyond that context.

Secondly, participants in Jones et al. [63] were asked to rate how much more attractive they found one target face compared with another, rather than indicate how attractive they found each target face individually. This may have prompted participants to think more carefully about their ratings and integrate additional sources of information–such as gaze cues–into the decision-making process. Kahneman [96] has suggested that “System 2” thinking, which involves slow, effortful, and deliberate thought processes, is more likely to be engaged when it is necessary to compare alternatives and make deliberate choices between options. Evaluation of individual faces in a context like the present study’s, on the other hand, has been characterised as a “System 1” process, involving rapid, effortless judgments that occur without conscious deliberation [59, 97].

Viewing the results described above through this theoretical lens can reconcile the apparently contradictory findings. When stimuli are neutral objects, gaze cues do not compete with an initial impression and are thus more likely to influence how those objects are evaluated. However, when stimuli are affectively valenced, like food or faces, people may tend to rely largely on their initial impressions such that the effect of emotional gaze cues from third parties is limited. Furthermore, human faces may evoke even stronger automatic evaluations than food due to humans’ highly social nature [98]. The exception may occur when they are prompted to update those impressions with additional information by a specific context or a need to choose between options.

One further possible reason for our failure to observe an effect of emotional gaze cues on face evaluations is the emotional expressions we used. Bayliss et al. [5] compared the effect of happy and disgusted expressions; in this study, our cue face models were asked to express liking and disliking. While this was arguably a more ecologically valid approach given that there was nothing inherently disgusting about our target stimuli (we acknowledge, of course, that one can feel disgust for another person without that other person actually having a disgusting appearance), it is possible that our cue faces’ emotional expressions were somewhat ambiguous or otherwise less strong than Bayliss et al.’s [5]. However, the replication of Bayliss et al.’s [5] central finding in Experiment 2 (albeit with a smaller effect size) suggests that it is unlikely that our stimuli were particularly problematic.

Our findings in relation to the effect of multiple cues contrast with what was reported by Capozzi et al. [57]. Again, there were important procedural differences between the present study and Capozzi et al. [57] that may have contributed to the divergent results. The first is that Capozzi et al.’s [57] multiple cue condition involved seven different cues, compared to three in this study. The second difference was the way in which the multiple cues were presented. In Capozzi et al. [57], different cue faces were presented individually over seven different trials. Here, all three cue faces were presented at once. This simultaneous presentation of multiple cue faces may have led participants to infer that the cue faces were not independent sources of information, which may have reduced their net influence. A third difference was that in Capozzi et al. [57] all the cue faces had relatively neutral expressions, with the result that the emotional expression of a single cue face may have appeared to the participants to be ambiguous. Multiple cue faces would therefore have been needed to provide an unambiguous signal. Conversely, in our study the expression of each cue face was deliberately chosen to be unambiguous which may have obviated the benefit of having multiple cue faces.

Because gender differences were not a focus of this study, we did not vary the gender of cue faces or recruit a balanced sample of participants. We note that the use of exclusively male cue faces and mostly female participants (the proportion of female participants ranged from a low of 72% in Experiment 2 to a high of 89% in Experiment 1) across each of the four studies may have contributed to our findings. However, it is not entirely clear what role gender might have played. A number of studies have shown that women respond more strongly to gaze cues than men when the dependent measure is reaction time, but there is no suggestion in the literature that this is modulated by the sex of the cue face. Bayliss et al. [70] investigated differences in gaze cueing as a function of both participant and cue face gender. In that study, female participants displayed stronger gaze cueing effects than males; however, there was no modulation of gaze cueing by the gender of the cue face. Alwall et al. [69] observed larger gaze cueing effects in female participants in a study in which only a female cue face was used. Deaner et al. [71] used all male cue faces and once again found that women showed larger gaze cueing effects than male participants, with the effect being particularly pronounced when the female participants were familiar with the male cue faces. Our findings with respect to gaze cueing of attention are largely in agreement with this research. Using mostly female participants, we observed strong effects of gaze cueing on reaction times in three of our four studies; and the one study in which this effect was marginal was the study with the smallest proportion of female participants (Experiment 2). It is of course possible that while gaze cues exert a stronger influence on the orientation of attention in women than men, the same relationship does not hold with respect to evaluations. To our knowledge there is no research addressing this question, and it may be worth pursuing in future work.

It is also important to acknowledge the difficulty of interpreting null results, even with (or, perhaps, because of) the added flexibility offered by Bayesian statistics [99]. While our Bayesian analyses suggest that the evaluations of faces are not susceptible to the influence of gaze cues, and that multiple, simultaneous gaze cues do not enhance the effect of gaze cues on evaluations, further evidence is needed to firm up these conclusions. It could be that our results apply only to our specific paradigm and may not generalize to different paradigms.

### Reaction times

Results of reaction time analyses were broadly consistent with the literature. With the exception of Experiment 2, participants were quicker to classify cued objects and target faces even though they knew that these gaze cues did not predict the location of target stimuli. Given the weight of evidence in both this study and the literature more broadly, the most plausible explanation for the non-significant effect of gaze cues on reaction time in Experiment 2 would appear to be Type II error. As in Bayliss et al. [5] and a number of other studies [27, 45, 46], the emotion of the cue face (or faces) did not appear to play a role in this gaze cueing effect. This was not a surprise given that cue faces did not display either of the emotions that have led to stronger gaze cueing effects in previous research (disgust and fear) [54–56].

### Conclusion

Previous research and theory suggest that gaze cues can affect how we evaluate both everyday objects and more significant aspects of our environment, such as other people. In the present study, however, there was no evidence that emotionally expressive gaze cues influenced evaluations of unfamiliar faces, nor was there evidence that the effect of gaze cues became more pronounced as the number of sources increased. Although our hypotheses were not supported, this study’s results are nonetheless important. Firstly, they identify the need for direct replication and systematic extension of previously reported effects in order to better understand their strength and boundary conditions. Secondly, the suggestion that gaze cues might have a stronger effect on affective evaluations when circumstances encourage System 2 thinking generates clear predictions that can be tested by modifying this study’s procedure. For example, the effect of gaze cues should be stronger when participants are required to compare stimuli rather than rate them individually. Finally, our reaction time data add further support to a growing consensus in the literature that only certain emotional expressions in certain experimental contexts lead to enhanced effects of gaze cues on the orientation of attention.

## Supporting Information

S1 Experiment 1 Dataset Raw data from Experiment 1 in Excel format. (XLSX)

S2 Experiment 2 Dataset Raw data from Experiment 2 in Excel format. (XLSX)

S3 Experiment 3 Dataset Raw data from Experiment 3 in Excel format. (XLSX)

S4 Experiment 4 Dataset Raw data from Experiment 4 in Excel format. (XLSX)
